# Depression among Infertile Women in Gaza Strip: Symptom Severity and Predictors

**DOI:** 10.1155/2021/6616489

**Published:** 2021-01-29

**Authors:** Aymen Elsous, Sae'd Abu El-Kass, Akram Salama, Mahmoud Radwan, Soha Abo-Eid, Suha Baloushah

**Affiliations:** ^1^Faculty of Medical Sciences, Israa University-Gaza, Gaza Strip, State of Palestine; ^2^Unit of Planning and Policy Formulation, Ministry of Health, Gaza Strip, State of Palestine; ^3^Department of Nursing, Faculty of Medical Sciences, Al Aqsa University, Gaza Strip, State of Palestine; ^4^Department of Nursing and Health Sciences, University College of Applied Sciences, Gaza Strip, State of Palestine; ^5^Department of Research, General Directorate of Nursing, Ministry of Health, Gaza Strip, State of Palestine; ^6^Department of Health Management and Economics, School of Public Health, Tehran University of Medical Science, International Campus, Tehran, Iran; ^7^Shifa Medical Complex, Palestinian Ministry of Health, Gaza Strip, State of Palestine; ^8^General Administration of Nursing, Palestinian Ministry of Health, Gaza Strip, State of Palestine; ^9^Department of Reproductive Health, School of Nursing and Midwifery, Tehran University of Medical Science, International Campus, Tehran, Iran

## Abstract

**Background:**

Mental disorders are expected for women suffering from infertility. Depression is a predictable consequence but requires more investigations and considerations. This study is aimed at determining the severity of depression symptoms and its predictors among infertile women in the Gaza Strip, Palestine.

**Materials and Methods:**

A cross-sectional study was conducted. Three hundred eighty-five infertile women participated and were selected by convenience sampling. The participated women were selected from three large and major in vitro fertilization treatment centers (Al Helo, Al Basma, and Hindawi). A validated Arabic version of the Beck Depression Inventory-II was used. Univariate and multivariate logistic regression was applied to determine potential predictors for depression symptoms, and *p* ≤ .05 was statistically significant.

**Results:**

The mean age of participated women was 29 ± 6.58 years, and the mean duration of marriage and infertility was 7.76 ± 5.31 and 5.43 ± 3.50 years, respectively. Half of the women had some form of depression symptoms (22.3%, 8.6%, and 10.6% showed to have mild depression, moderate, and severe depression symptoms, respectively). Predictors of depression symptoms were as follows: duration of marriage (Wald test: 10.493; CI95%: 0.248-0.774), at least one abortion (Wald test: 21.233; CI95%: 1.863–4.528), primary infertility (Wald test: 6.666; CI95%: 1.148–2.742), and husband cause of infertility (Wald test: 10.878; CI95%: 0.800–0.982).

**Conclusion:**

Infertility affects the various aspect of women's life. Psychological intervention including counselling, support, and therapy would be necessary to limit the consequences of infertility. Such interventions could be implemented in infertility treatment clinics.

## 1. Introduction

Infertility is the inability of couples to achieve pregnancy and to have children after at least one year or more of organized sexual intercourse without protection methods [1]. Infertility is not only a physical dysfunction or organ failure, but it affects all aspects of couples' life which require further care and attention [2].

Infertility could be a psychological threat and one of the most important lifetime crises and socially stigmatized for infertile women [3]. In Arabic cultures, infertile women may face more psychological disturbances than men because they feel accountable for the inability to get pregnant or provide childbearing [4]. Depression symptoms might exist however unnoticed, and physicians usually give attention to the physical part of treatment away from the psychological aspect [5].

Depressed patients have mood disorders, loss of enjoyment in routine activities, feelings of low self-esteem, sleep disturbance, lethargy, and flight of ideas [6]. Psychological researches have shown that infertility may lead to depression or a raise in symptoms if previously existed in person [7]. Begum and Hasan found a high prevalence of depression among infertile individuals compared to fertile ones [8].

The relationship between infertility and depression was reported widely [1]. Infertile women showed to have much more depression when their partners suffered from impotence. Ozturk et al. reported a 29.2% spread of depression symptoms among infertile women with impotent males compared to 4.3% among impotent males [1].

According to the World Health Organization (WHO), approximately 60-80 million individuals have a type of infertile couples worldwide [9]. Consequently, nearly 40% of individuals who seek infertility treatment are unable to achieve pregnancy [10]. In Palestine, there is no accurate data about the prevalence of infertility among couples. However, based on the Palestinian Central Bureau of Statistics (PCBS), the prevalence of infertile women in Palestine was reported to be 8.4% in the West Bank and 8.3% in Gaza Strip [11]. Infertility is a psychosocial burden for childless women who have no history of pregnancy and feel low self-esteem accompany by social pressure from their husbands, their families, and husbands' families, therefore resulting in social isolation and feeling stigmatized [12]. This study is aimed at determining the severity of depression symptoms and exploring the predictors among infertile women in the Gaza Strip, Palestine.

## 2. Materials and Methods

### 2.1. Study Design and Setting

This present study is a descriptive-analytic cross-sectional study. The cross-sectional study helps the researcher to take a snapshot of the current health problem under investigation. The study was conducted at three major IFV centers in Gaza city from January to December 2019. The centers were Al Helo, Al Basma, and Al Hindawi infertility clinics. The study was conducted among infertile women who sought IVF treatment in the abovementioned selected infertility clinics.

### 2.2. Study Sample and Sampling

A nonprobability convenient sample was applied to select the required participants. The present study included 385 participants with margin of error 5% and confidence level 95%. Women who were diagnosed with primary or secondary infertility agreed to participate in our study voluntarily.

### 2.3. Study Instrument

The translated and valid Arabic version of the Beck Depression Inventory (BDI) scale, an assessment tool to measure severity of depression symptoms, was used [13]. The BDI includes 21 items in which each item describes a specific behavioral, emotional, and somatic manifestation of depression. The BDI items cover sadness, pessimism, sense of failure, dissatisfaction, guilt, the expectation of punishment, self-dislike, self-accusations, suicidal ideas, crying, irritability, social withdrawal, indecisiveness, body image change, work retardation, insomnia, fatigability, anorexia, weight loss, somatic preoccupation, and loss of libido [14]. The score of the BDI is based on a 4-point Likert scale. It ranges from “0” that indicates no depression symptoms to “4” for severe depression symptoms. Therefore, the overall scale score ranges from 0 to 63. The BDI scores are classified as no depression symptoms (scores 0-13), mild (14-19), moderate (20-28), and severe (29-63) [15]. Sociodemographic and infertility history data including age, residency, education, employment, duration of the marriage, polygamy husband, type of infertility, duration of infertility, IVF times, and infertility caused were also gathered. Data was gathered through the exit, face to face-based interviews. Pilot interviews were conducted with 20 infertile female patients to ensure clarity of questions. The results of the pilot were excluded from the final data collection. Three well-trained data collectors were recruited to gather the data from the eligible infertile women in the three IVF centers.

### 2.4. Data Analysis

Data were analyzed using the Statistical Package for Social Sciences Software Version 22. Continuous variables were presented to inform of mean and standard deviation. Frequency and percentage were presented for categorical variables. Bivariate analysis was conducted to select independent variables for multivariate logistic regression. We aimed to take a wide number of independent variables, and, therefore, independent variables with *p* < .250 were chosen accordingly because the frequently used *p* ≤ .05 usually fails to capture significant variables [16]. Multivariate regression analysis for potential predictors of depression symptoms in infertile women was performed, and *p* ≤ .05 was considered statistically significant.

### 2.5. Ethical Approval

The study was approved by the Palestinian health research counseling, HELSINKI committee (PHRC/HC/277/17). Also, the invited participant signed on consent form before their participation. Anonymity and voluntary participation were also ensured.

## 3. Results

Three hundred eighty-five infertile women participated. The women's mean age ± SD was 29 ± 6.58 years, duration of marriage was 7.76 ± 5.31 years, IVF trial times was 1.82 ± 1.88, and duration of infertility was 5.43 ± 3.50 years. Sixty percent (227/385) completed at least the university level.

Majority were housewives (78.4%), and 34.3% of women infertility was related to their husbands. Sociodemographic and Obst/Gyn characteristics of the study participants are summarized in Table 1. According to BDI, 22.3% (86/350) showed to have mild depression symptoms, and 8.6% (33/350) and 10.6% (41/350) had moderate and severe depression symptoms, respectively. Around half of women (49.4%) showed no symptoms of depression. The mean score of depression symptoms increased when the level of education improved. Depression symptoms increased with higher education although there was no statistical significance (*p* = 0.271). Duration of marriage (*p* < 0.001) and type of infertility (*p* < 0.05) tend to have influence on the emotional status. Longer marital years and primary infertility have a positive influence on the occurrence of depression symptoms. Moreover, depression symptoms increased when infertility cause was linked to husbands or an unknown cause (*p* < .012). The frequency of depression symptoms increased when the duration of infertility is less than 5 years; however, it is not significant (*p* = .985). Depression symptoms are more among women who experienced at least two IVF attempts (*p* < 0.5). Unemployed women reported more depression symptoms than employed women but it is not significant (*p* = .981) (Table 2).

Findings from logistic regression analysis showed duration of marriage (>6 years) (Wald test: 10.493; CI95%: 0.248-0.774; *p* = .001), at least one abortion (Wald test: 21.233; CI95%: 1.863–4.528; *p* < .0001), primary infertility (Wald test: 6.666; CI95%: 1.148–2.742; *p* = .01), and husband cause of infertility (Wald test: 10.878; CI95%: 0.800–0.982; *p* < .05) were predictors for depression symptoms of infertile women (Table 3).

## 4. Discussion

This study was conducted to determine the distribution of depression symptoms among infertile females. According to our society's norms and culture, having a child is an important issue in marital life, and inability to have it would create challenges for infertile couples, particularly females. Infertile females are deprived of their important maternal role and are ultimately threatened by mental disorders. To the best of our knowledge, this is the first study addresses depression symptoms in infertile women. A growing body of evidence emphasized the psychological aspect of infertility in women [1, 6, 10, 17–23]. Infertility is a source of stress for couples who are unable to conceive, and depression was most commonly reported, and its incidence varied among societies and countries. However, a study found the occurrence of depression in infertile individuals is no more common than in the entire population [24]. The Depression Beck Inventory is a screening or an assessment tool characterized by good sensitivity but low specificity.

In our study, 50.6% of infertile women had some form of depression symptoms. This finding is similar to reports from Nigeria and Saudi Arabia [6, 22] which were 52.7% and 53.8%, respectively. Other studies revealed low severity [25, 26]. Differences in severity rate could be attributed to using of various instruments to assess depression symptoms. Some have used the Patient Health Questionnaire, the Beck Depression Inventory (BDI), the General Health Questionnaire (GHQ), or the Hospital Anxiety and Depression Scale (HADS).

Husband infertility was a significant predictor for depression symptoms in women. This is in line with previous studies [18, 27–29]. Men in Middle Eastern countries practice their family role as powerful virile using reproduction; thus, infertility is a challenging and threatening problem that may result in psychological distress to their counterpart women. These psychological distresses are displacing women because they are submissive and dependent on males. Males' infertility may also result in low sexual self-esteem and performance, and if emotional connection, in terms of sexual intercourse, failed to achieve its purpose, couples might be divided or isolated. Thus, women remain under stress for a prolonged duration. Savadzadeh and Madadzadeh [29] stated that if males' capabilities failed to get their partners pregnant, a devastating emotional sequence, including depression, is expected for couples however is much more among wives. By and large, male infertility factors lead to a psychological burden for both men and women; however, it is much more in women [30].

Duration of infertility was not a predictor of depression symptoms. This is in line with findings from Iran [19, 27], Nigeria [26], and Japan [31]. In return, infertility duration showed to affect the occurrence of depression in the first three years of infertility; however, depression symptoms subsequently decreased with time [32]. A possible explanation could be attributed to adjustment of infertile women to infertility, sharing a problem with medical professionals, and the hope for successful trials under new technologies and techniques which give hope for pregnancy. Moreover, the desensitization model presented by Kopitzke et al. [33] could also be applied. The model describes an individual's stress response to initial diagnosis and his/her coping with prolonged exposure to stressful situations and becomes desensitized.

In our study, we found no significant relationship between age and depression symptoms. In line with studies' findings [21], the female age was not a predictor for the occurrence of depression symptoms. This is in contrast to the reports of Alhassan et al. [17], Al-Homaidan [6], and Awoyinka and Ohaeri [34] from Ghana, Saudi Arabia, and Nigeria, respectively. We do speculate that young females expose to psychological and social pressure similar to their older counterparts which hide age-related symptoms of depression.

Duration of marriage was a significant predictor for depression symptoms similar to the previous studies [24, 25]. However, this is not in line with Oladeji and OlaOlorun [21]. A possible explanation could be attributed to different geographical locations and societies that held different beliefs and norms and to social or partner support.

Abortion was found to be a significant predictor for depression symptoms among infertile women. This result is in agreement with the findings of Kolte et al. [35], Zamani et al. [36], and Adib-Rad et al. [37]. Similarly, Kagami et al. [38] used the Beck Depression index and found significantly higher levels of depression symptoms among women as compared with men. Recurrent abortion is a serious point for infertile women, and it is not easy to cope or recover spontaneously. For sure, they require kind of support, care, and interventions to help them to adjust after pregnancy loss. Moreover, we think that infertile women after spontaneous abortion lose hope to become mothers and thus may produce a source of internal psychological pressure and guilty feeling.

Type of infertility is another predictor of depression symptoms. This result is similar to the previous publish results from a survey about depression among infertile women in Ghana [17], Saudi Arabia [6], and in Iraq [39]. Having a child is very important to the Palestinian community. Childbearing women have higher marital satisfaction and social value among their family members. Being unable to conceive adds psychosocial burden over the infertile woman's life. Infertility affects infertile women's marriage stability and psychological well-being as it is proven in our research result.

The study has many limitations; firstly, the study approached only infertile females who seek IVF treatment; however, there were still infertile women who are economically unaffordable to seek treatment, and we believe that depression symptoms could be much higher. We do recommend further studies on this part of the population. Secondly, the conservative culture of the Palestinian community makes it hard sometimes to ask questions about infertility and sexual function. Thirdly, the nature of the cross-sectional design limits the establishment of causal inferences with study variables. Fourthly, using of self-rating questionnaire limited the in-depth exploration of psychological distresses and depression symptoms. The advantage of the study is that findings reflect the characteristics of the general population in infertile women because most famous infertility treatment clinics, including the selected center in our study, are located in Gaza city.

Infertility affects different aspects of peoples' life including the psychological part. Symptoms of depression are presented in half of the infertile females. Females' age, duration of infertility, and IVF attempts are not predictors for depression. However, the duration of the marriage, abortion times, and male factor infertility are predictors with high significance. Ignorance of psychological aspects of infertility may threaten the continuity of couples' marriage and prolong treatment courses. Thus, psychological intervention including counselling, support, and therapy would be necessary to prevent or overcome the implications of infertility. Such interventions could be implemented in infertility treatment centers or through nongovernmental organizations.

## Figures and Tables

**Table 1:** Baseline characteristics of participated women.

Serial	Variable	*n*	%
1	Age (M ± SD: 29 ± 6.58)		
	≤30 years	254	66.0
	>30	131	34.0

2	Living place		
	North	72	18.7
	Gaza	182	47.3
	Middle area	62	16.1
	South	69	17.9

3	Education		
	Illiterate	25	6.5
	Up to sec. school	133	34.5
	≥ university	227	59.0

4	Employment		
	Have job	83	21.6
	Housewife	302	78.4

5	Duration of marriage (M ± SD: 7.76 ± 5.31)		
	≤6 years	188	48.8
	>6	197	51.2

6	Polygamy husband		
	Yes	47	12.2
	No	338	87.8

7	Type of infertility		
	Primary	242	62.9
	Secondary	143	37.1

8	Duration of infertility (M ± SD: 5.43 ± 3.50)		
	≤5 years	219	56.9
	>5	162	42.1

9	IVF times (M ± SD: 1.82 ± 1.88)		
	≤2 times	274	71.2
	>2	106	27.5

10	No. of abortion (M ± SD: 0.74 ± 1.31)		
	Zero	249	64.7
	≥1	136	35.3

11	Infertility cause		
	Wife cause	54	14.0
	Husband cause	132	34.3
	Both	78	20.3
	Unknown	121	31.4

**Table 2:** Comparison of severity of depression symptoms with regard to participants' characteristics.

Serial	Variables	Level of depression				
		No depression	Mild depression	Moderate depression	Severe depression	*p*
1	Age					
	≤30 years	131	58	15	25	.061
	>30	59	28	18	16	

2	Living place					
	North	38	13	8	6	
	Gaza	91	44	9	20	.278
	Middle area	25	14	10	9	
	South	36	15	6	6	

3	Education					
	Illiterate	6	8	4	4	
	Up to sec. school	64	28	9	14	.271
	≥ university	120	50	20	23	

4	Duration of marriage					
	≤6 years	108	39	14	10	.001
	>6	82	47	19	31	

5	Polygamy husband					
	Yes	21	9	4	7	.716
	No	169	77	29	34	

6	Duration of infertility					
	≤5 years	107	50	18	24	.985
	>5	79	36	15	17	

7	IVF times					
	>2 times	142	64	22	22	.055
	≤2	46	22	11	18	

8	Infertility cause					
	Wife cause	21	18	5	6	
	Husband cause	31	27	8	6	.012
	Both	30	19	10	11	
	Unknown	58	22	10	18	

9	Type of infertility					
	Primary	129	54	18	15	0.02
	Secondary	61	32	15	26	

10	Employment					
	Have a job	43	18	8	9	.981
	Housewife	147	68	25	32	

11	No. of abortion					
	Zero	142	50	17	14	<.0001
	≥1	48	36	16	27	

**Table 3:** Multivariate logistic regression analysis for potential predictors to depression.

Serial	Variable	B	S.E.	Wald	*p* value	Exp (B)	95% CI lower-upper
1	Age						.610–1.787
	≤30 years					1	
	>30	-.172	.107	2.565	.109	.842	

2	Duration of marriage						.248-.774
	≤6 years					1	
	>6	.707	.218	10.493	.001	2.028	

3	No. of abortion						1.863–4.528
	Zero					1	
	≥1	-1.060	.230	21.233	.0001	2.885	

4	IVF times						.533–1.583
	≤2 times					1	
	>2	-.168	.108	2.418	.120	.846	

5	Infertility cause	-.172	.107	2.565	.109		.800–.982
	Wife cause	.769	.233	10.878	.001	.842	
	Husband cause	-.575	.270	4.540	.033	2.157	
	Both					.563	
	Unknown					1	

6	Type of infertility	.573	.222	6.666	.010		1.148–2.742
	Primary					1.774	
	Secondary					1	
	Constant	.460	.246	3.509	.031	1.584	

## Data Availability

The data used to support the findings of this study are available from the corresponding author upon request.
